# Presentation, management, and outcomes of older compared to younger adults with hospital-acquired bloodstream infections in the intensive care unit: a multicenter cohort study

**DOI:** 10.1007/s15010-024-02304-y

**Published:** 2024-06-13

**Authors:** Ili Margalit, Dafna Yahav, Tomer Hoffman, Alexis Tabah, Stéphane Ruckly, François Barbier, Pierre Singer, Jean-François Timsit, Virginie Prendki, Niccolò Buetti

**Affiliations:** 1 Infectious Diseases Unit, Sheba Medical Center, Ramat-Gan, Israel; 2 Faculty of Medical & Health Sciences, Tel Aviv University, Ramat Aviv, Tel Aviv, Israel; 3 Intensive Care Unit, Redclife Hospital, Brisbane, QLD Australia; 4 Queensland University of Technology, Brisbane, QLD Australia; 5 Faculty of Medicine, The University of Queensland, Brisbane, QLD Australia; 6 IAME UMR 1137, INSERM, Université de Paris, 75018 Paris, France; 7 ICUREsearch, Biometry, 38600 Fontaine, France; 8 Service de Médecine Intensive‑Réanimation, Centre Hospitalier Régional d’Orléans, 14, Avenue de L’Hôpital, 45100 Orléans, France; 9 Department of General Intensive Care and Institute for Nutrition Research, Rabin Medical Center, Beilinson Hospital, Petah Tikva, Israel; 10 Medical and Infectious Diseases Intensive Care Unit, AP-HP, Bichat-Claude Bernard University Hospital, 46 Omdurman Maternity Hospital Rue Henri Huchard, 75877 Paris, France; 11 Division of Internal Medicine for the Aged, Department of Rehabilitation and Geriatrics, Geneva University Hospitals, Geneva, Switzerland; 12 Division of Infectious Diseases, Geneva University Hospitals, Geneva, Switzerland; 13 Infection Control Programme and World Health Organization Collaborating Center, Geneva University Hospitals and Faculty of Medicine, Geneva, Switzerland

**Keywords:** Bacteremia, Elderly, ICU, Mortality, Nosocomial Infections

## Abstract

**Purpose:**

Older adults admitted to the intensive care unit (ICU) usually have fair baseline functional capacity, yet their age and frailty may compromise their management. We compared the characteristics and management of older (≥ 75 years) versus younger adults hospitalized in ICU with hospital-acquired bloodstream infection (HA-BSI).

**Methods:**

Nested cohort study within the EUROBACT-2 database, a multinational prospective cohort study including adults (≥ 18 years) hospitalized in the ICU during 2019–2021. We compared older *versus* younger adults in terms of infection characteristics (clinical signs and symptoms, source, and microbiological data), management (imaging, source control, antimicrobial therapy), and outcomes (28-day mortality and hospital discharge).

**Results:**

Among 2111 individuals hospitalized in 219 ICUs with HA-BSI, 563 (27%) were ≥ 75 years old. Compared to younger patients, these individuals had higher comorbidity score and lower functional capacity; presented more often with a pulmonary, urinary, or unknown HA-BSI source; and had lower heart rate, blood pressure and temperature at presentation. Pathogens and resistance rates were similar in both groups. Differences in management included mainly lower rates of effective source control achievement among aged individuals. Older adults also had significantly higher day-28 mortality (50% versus 34%, *p* < 0.001), and lower rates of discharge from hospital (12% versus 20%, *p* < 0.001) by this time.

**Conclusions:**

Older adults with HA-BSI hospitalized in ICU have different baseline characteristics and source of infection compared to younger patients. Management of older adults differs mainly by lower probability to achieve source control. This should be targeted to improve outcomes among older ICU patients.

**Supplementary Information:**

The online version contains supplementary material available at 10.1007/s15010-024-02304-y.

## Introduction

The proportion of critically ill older adults admitted to intensive care unit (ICU) is constantly increasing. Alongside, incidence of hospital-acquired bloodstream infections (HA-BSI) is highest among older adults [1].

As aging is associated with changes in organ function, comorbidities, reduced mobility and cognition, the management of elderly patients in the ICU setting is challenging, with poorer outcomes. Older age, particularly > 75 years, was reported as a significant independent risk factor for mortality in ICU in general, and specifically among patients with severe sepsis [2, 3].

It was suggested that differences in management of infection between older and younger patients may exist, including lower rates of imaging studies, infectious diseases consultation, and/or surgical/drainage procedures [1]. However, data from large international cohorts including low- and middle-income countries are scarce. Moreover, frailty assessment has been advocated for triage and management purposes of older adults in the ICU. Even though ICU admissions of older adults is biased towards high functioning individuals at baseline, unrecognized frailty frequently reveals itself over the ICU course [4]. This may lead to changes in patients’ management by limiting some aspects of care [5]. Accordingly, the decision to forgo life-sustaining therapies while hospitalized in ICU is also more prevalent among older adults [6].

To assess prognosis of ICU-admitted older adults, it is imperative to determine whether management differences exist in comparison to younger counterparts.

We hypothesized that though accepted to be hospitalized in the ICU, older adults are managed differently from younger patients. Therefore, in this study we aimed to describe and compare the presentation and management, as well as 28-day mortality of two groups of patients hospitalized in the ICU and treated for HA-BSI, according to their age, using a cut-off of 75 years.

## Methods

### Study design and population

This cohort study was nested within the EUROBACT-2 database, a multinational prospective cohort study of adults (≥ 18 years) hospitalized in ICU, who developed HA-BSI, either after hospital admission or during ICU stay. HA-BSI was defined as ≥ 1 positive blood culture that was first sampled > 48 h following hospital admission. A total of 2600 individuals were recruited from 333 ICUs in 52 countries between 2019 and 2021. In cases of multiple HA-BSIs, only the first episode was included. Further details are provided in the supplement and elsewhere [7].

For the current study, we included only ICUs that recruited at least one old adult. We classified the study population into two age groups: older (≥ 75 years) and non-elderly individuals (< 75 years) and described their baseline comorbidities, characteristics of the infection, management, and outcomes. We compared these two subpopulations in terms of infection characteristics (clinical signs and symptoms, source, and microbiological data); management (imaging, source control, antimicrobial therapy), and 28-day mortality.

### Definitions

Empirical therapy was defined as adequate whenever an appropriate antimicrobial agent (i.e., in-vitro covering antibiotics according to later antimicrobial susceptibility results) was administered within 24 h from index blood culture collection.

Source control was classified according to the treating physicians in participating centers, as either attempted, achieved; attempted not achieved; or not attempted.

Functional limitation was defined as restriction of any degree (mild, moderate, or severe) to perform daily activities stemming from chronic illnesses or conditions.

### Statistical analysis

We compared older adults to non-elderly individuals using univariate logistic regression models through generalized estimating equations, accounting for a possible center effect.

Sensitivity analyses were performed by exclusion of individuals for whom a decision to avoid life-sustaining therapies was taken, by exclusion of individuals who were admitted with COVID-19, and by stratifying the cohort according to the median Human Development Index (HDI) (greater or ≤ than HDI = 0.85). We also performed an additional analysis through classifying the cohort according to baseline physical capacity status (any functional limitation vs none).

All analyses were performed using IBM SPSS Statistics, version 28.0 (IBM Corp., Armonk, NY, USA). For all analyses, *p* < 0.05 was considered statistically significant.

### Ethical aspects

The EUROBACT-2 trial was initially approved by the Research Ethics Committee of Royal Brisbane & Women’s Hospital, Queensland, Australia (LNR/2019/QRBW/48376). The study was further approved at each participating site according to national and/or local regulations.

## Results

**Fig. 1:**
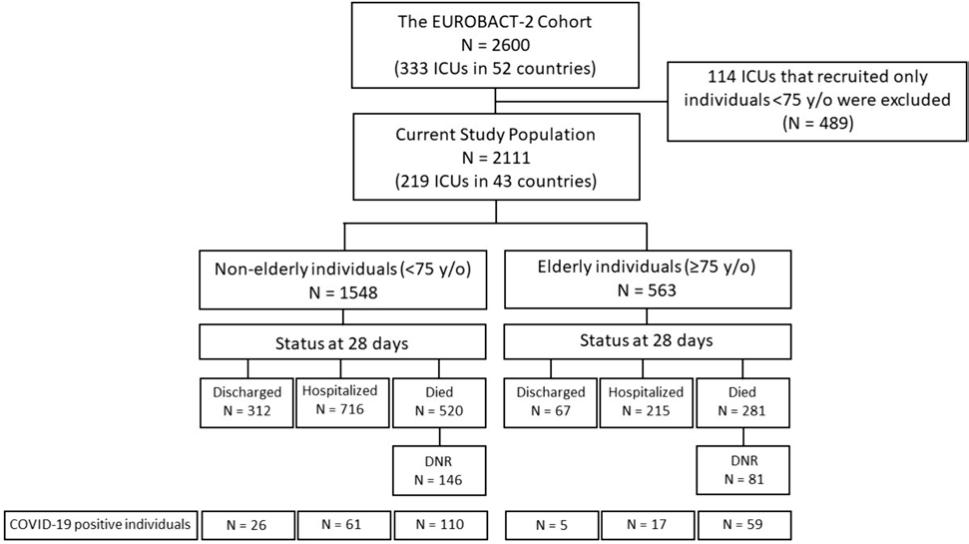
Study flow diagram. *DNR* do not resuscitate, *ICUs* intensive care units, *y/o* years old

Of the 333 ICUs from 52 countries comprising the EUROBACT-2 cohort, 219 ICUs from 43 countries reported on at least one older adult (≥ 75 years old). Accordingly, a total of 2111 individuals hospitalized in ICU and diagnosed with HA-BSI were included in the current study (Fig. 1). Of these, 1577 (75%) were from 142 (65%) ICUs in countries located within the WHO European Region. The median HDI of participating countries was 0.85 (IQR 0.76, 0.93), and a total of 1183 (56%) individuals were reported from ICUs in countries with an HDI of the higher median (Supplementary Table S1).

Of the total cohort, 563 (27%) were older adults (≥ 75 years old).

### Comorbidities and functional status

Older adults had higher Charlson comorbidity index (median 2 [IQR 1.4] vs 1 [IQR 0.4], *p* < 0.001) and generally more comorbidities, however non-elderly individuals had higher proportions of severe liver disease (4% vs 1%, *p* = 0.003), hematological malignancy (7% vs 4%, *p* = 0.020), and transplant recipients (4% vs 0.2%, *p* = 0.002). Older adults exhibited higher proportions of baseline functional limitation, and only 28% of whom were completely independent compared to 45% of the non-elderly individuals (*p* < 0.001). Younger individuals had slightly higher average BMI (median 26.2 [IQR 23.5, 30.2] vs 26.2 [23.5, 29.7], mean 27.5 [SD 7.4] vs 26.7 [5.4], *p* = 0.015).

Altogether, 278 (13%) of the cohort were admitted due to COVID-19, with similar proportions between both age groups (13% vs 14%, *p* = 0.319). (Table 1).

**Table 1:** Characteristics of 2111 individuals diagnosed with HA-BSI, according to age category, adjusted for center

	18–75 years N = 1548 (73%)	≥ 75 years N = 563 (27%)	Adjusted* p*^a^
Age (years), median (IQR)	60 (49,68)	80 (77,84)	NA
Female gender, N (%)	550 (35.5)	219 (38.9)	0.155
Body mass index (BMI), median (IQR)	26.2 (23.5,30.2)	26.2 (23.3,29.7)	0.015
Comorbidities			
Charlson score, median (IQR)	1 (0,3.75)	2 (1.00,4.00)	< 0.001
COPD (moderate or severe), N (%)	225 (14.5)	123 (21.8)	< 0.001
Heart failure (NYHA classes 3–4), N (%)	127 (8.2)	110 (19.5)	< 0.001
Myocardial infarction, N (%)	132 (8.5)	73 (13.0)	0.002
Peripheral vascular disease, N (%)	87 (5.6)	59 (10.5)	< 0.001
Cerebrovascular disease, N (%)	151 (9.8)	89 (15.8)	< 0.001
Dementia, N (%)	26 (1.7)	76 (13.5)	< 0.001
Hemiplegia, N (%)	40 (2.6)	23 (4.1)	0.075
Diabetes mellitus, N (%)	408 (26.4)	174 (30.9)	0.039
Moderate renal disease, N (%)	135 (8.7)	79 (14.0)	< 0.001
Hemodialysis, N (%)	80 (5.2)	22 (3.9)	0.234
Connective tissue disease, N (%)	40 (2.6)	10 (1.8)	0.283
Peptic ulcer disease, N (%)	47 (3.0)	27 (4.8)	0.054
Severe liver disease, N (%)	55 (3.6)	5 (0.9)	0.003
HIV, N (%)	8 (0.5)	1 (0.2)	0.313
Solid malignancy, N (%)	133 (8.6)	76 (13.5)	< 0.001
Metastatic solid tumor, N (%)	94 (6.1)	37 (6.6)	0.674
Hematological malignancy, N (%)	106 (6.8)	23 (4.1)	0.020
Transplant recipients, N (%)	57 (3.7)	1 (0.2)	0.002
Corticosteroid therapy, N (%)	87 (5.6)	21 (3.7)	0.083
Functional status before hospitalization			
No limitation, N (%)	695 (44.9)	156 (27.8)	< 0.001
Mild to moderate limitation, N (%)	529 (34.2)	240 (42.7)	< 0.001
Serious but not incapacitation restriction, N (%)	212 (13.7)	106 (18.9)	0.004
Severe restriction including bedridden, N (%)	111 (7.2)	60 (10.7)	0.010
Admitted due to COVID-19	197 (12.7)	81 (14.4)	0.319
Hospital-acquired Bloodstream infection			
Acquisition site			0.755
ICU, N (%)	1225 (79.1)	442 (78.5)	
Non-ICU, N (%)	323 (20.9)	121 (21.5)	
Duration of hospital stay prior to HA-BSI detection, median (IQR)	14 (8,25)	13 (8,25)	0.931
Duration of ICU stay prior to HA-BSI detection, median (IQR)	7 (2,15)	6 (1,14)	0.550
Most likely source of HA-BSI			
Respiratory, N (%)	387 (25.0)	157 (27.9)	0.180
Primary, N (%)	260 (16.8)	112 (19.9)	0.099
Catheter-related, N (%)	430 (27.8)	122 (21.7)	0.005
Intra-abdominal, N (%)	242 (15.6)	78 (13.9)	0.314
Urinary, N (%)	103 (6.7)	50 (8.9)	0.082
Other, N (%)	126 (8.1)	44 (7.8)	0.809
Antimicrobial therapy during the 7 days prior to bloodstream infection, N (%)	1123 (72.7)	408 (72.6)	0.968
Ventilatory requirements during BSI			
Invasive mechanical ventilation, N (%)	1066 (68.9)	384 (68.2)	0.773
Non-invasive ventilation, N (%)	91 (5.9)	37 (6.6)	0.555
High flow oxygen, N (%)	101 (6.5)	36 (6.4)	0.914
Low flow or no need for oxygen supplementation, N (%)	290 (18.7)	106 (18.8)	0.961
SOFA, median (IQR)	8 (5,11)	8 (6,12)	0.073
Glasgow Coma Scale, median (IQR)	13 (8,15)	12 (8,15)	0.059
*Vital signs at time of BSI presentation*			
Maximal heart rate (beats per minute), median (IQR)	113 (97,130)	110 (94,126)	0.019
Minimal mean arterial pressure (mmHg), median (IQR)	66 (58,75)	65 (56,74)	0.012
Maximal body temperature (℃), median (IQR)	38.1 (37.2,38.8)	37.6 (36.9,38.4)	< 0.001
Hypothermia, N (%)	240 (15.6)	89 (15.9)	0.876
Mental state, N (%)			
Conscious and normal neurological status	459 (29.8)	164 (29.3)	0.804
Hyporeactive delirium	128 (8.3)	55 (9.8)	0.282
Mixed delirium	57 (3.7)	13 (2.3)	0.122
Hyperreactive delirium	33 (2.1)	16 (2.9)	0.341
Comatose/unconscious, with ongoing sedation	653 (42.5)	216 (38.6)	0.110
Comatose/unconscious, without ongoing sedation	208 (13.5)	96 (17.1)	0.038
* Laboratory parameters at time of BSI presentation*			
Maximal C-related protein (mg/L), median (IQR)	119 (45,212)	122 (45,215)	0.896
Maximal procalcitonin (ng/mL), median (IQR)	2.4 (0.6,10.1)	1.8 (0.5,10.2)	0.473
Maximal creatinine (mg/dL), median (IQR)	2.0 (0.8,4.7)	1.8 (1.0,4.0)	0.083
Minimal platelet count (1000/µL), median (IQR)	186 (104,281)	189 (109,274)	0.647
Maximal white blood cell count (1000/µL), median (IQR)	12.8 (8.6,19.0)	14.3 (9.7,20.5)	0.180
Microbiology			
Causative pathogen			
Gram-negative bacteria, N (%)			0.806
* Enterobacterales* ^b^, N (%)	476 (30.7)	179 (31.8)	
* Pseudomonas aeruginosa*, N (%)	104 (6.7)	41 (7.3)	
* Acinetobacter baumannii*, N (%)	177 (11.4)	60 (10.7)	
Gram-positive bacteria, N (%)			0.591
* Staphylococcus aureus*, N (%)	134 (8.7)	43 (7.6)	
Coagulase-negative *Staphylococci*, N (%)	132 (8.5)	39 (6.9)	
* Enterococcus* species, N (%)	122 (7.9)	54 (9.6)	
Candida, N (%)	112 (7.2)	50 (8.9)	
Other, N (%)	116 (7.5)	34 (6.0)	
Polymicrobial, N (%)	175 (11.3)	63 (11.2)	
Resistance pattern of the isolate ^c^			
Difficult to treat gram negative bacteria, N (%)	173 (11.2)	73 (13.0)	0.257
Methicillin-resistant *Staphylococcus aureus*, N (%)	44 (2.8)	21 (3.7)	0.298
Methicillin-resistant *Staphylococcus epidermidis*, N (%)	102 (6.6)	30 (5.3)	0.291
Vancomycin-resistant *Enterococcus*, N (%)	15 (1.0)	10 (1.8)	0.135

*BSI* bloodstream infection, *CI* confidence interval, *COPD* Chronic obstructive pulmonary disease, *HIV* human immune deficiency virus, *ICU* intensive care unit, *IQR* interquartile range (first and third quartiles), *NYHA* New York Heart Association, *SAPS* Simplified Acute Physiology Score

^a^Calculated using logistic regression models adjusted for center effect through generalized estimation equations

^b^*Citrobacter* spp., *Enterobacter* spp., *Escherichia coli*, *Hafnia alvei*, *Klebsiella* spp., *Morganella morganii*, *Pantoea*, *Proteus* spp., *Providencia*, *Serratia* spp.

^c^Eleven individuals were diagnosed with polymicrobial bacteremia of with two resistant pathogens: 2 individuals with both carbapenem-resistant *Acinetobacter baumannii* (CRAB) and difficult to treat *Klebsiella* sp., 3 individual with CRAB and meticillin-resistant *Staphylococcus epidermidis* (MRSE), 1 individual with CRAB and meticillin-resistant *Staphylococcus aureus* (MRSA), and five more individuals with difficult to treat *Klebsiella pneumonia* coinfected with MRSE (N = 2), MRSA (N = 1), vancomycin resistant *enterococcus faecium* (N = 1), and difficult to treat *Providencia* sp. (N = 1)

### Patient characteristics at bloodstream infection diagnosis

The source of HA-BSI slightly differed between the sub-populations (*p* = 0.021). Older adults had lower proportions of catheter-related BSI (22% vs 28%, *p* = 0.005). Additionally, older adults tended to have higher proportions of respiratory (28% vs 25%, *p* = 0.180) or urinary (9% vs 7%, *p* = 0.082) source, and primary bacteremia (20% vs 17%, p = 0.099). Younger individuals tended to have higher proportions of intra-abdominal source (16% vs 14%, *p* = 0.314). Older adults had lower maximal heart rate (median 110 [IQR 94, 126] vs 113 [97, 130] beats per minute, *p* = 0.019), minimal mean arterial pressure (median 65 [IQR 56, 74] vs 66 [58, 75] mmHg, *p* = 0.012) and maximal body temperature (median 37.6 [IQR 36.9, 38.4] vs 38.1 [37.2, 38.8] °C, *p* < 0.001), though SOFA scores, blood parameters and ventilatory requirement were non-significantly different. However, a higher proportion of older adults were presented with non-sedative coma (17% vs 14%, *p* = 0.038).

The causative pathogens and resistance patterns were similar between the two sub-populations (Table 1).

### Management of bloodstream infection

Imaging was performed similarly for both groups, except for MRI that was performed less frequently among older adults (1.6% vs 3.2%, *p* = 0.049). The latter were also less likely to receive adequate antibiotic treatment within 24 h, although this finding did not reach statistical significance (49% vs 53%, *p* = 0.051).

Among older patients, source control was less often indicated (44% vs 53%, *p* < 0.001), and, when attempted, was less frequently effective (77% vs 83%, *p* = 0.017). Additionally, Extracorporeal membrane oxygenation (ECMO) was also significantly less used among older adults (0.2% vs 1.9%, *p* = 0.020) (Table 2).

**Table 2:** Management of HA-BSI among 2111 individuals, according to age category, adjustments for center

	18–75 years N = 1548 (73%)	≥ 75 years N = 563 (27%)	Adjusted* p*^a^
Imaging and additional diagnostic tests			
CT, N (%)	621 (40.1)	232 (41.2)	0.651
MRI, N (%)	50 (3.2)	9 (1.6)	0.049
US, N (%)	338 (21.8)	114 (20.2)	0.432
PET-CT, N (%)	15 (1.0)	6 (1.1)	0.843
Cardiothoracic echocardiography, N (%)	369 (23.8)	127 (22.6)	0.540
Cardio-esophageal echocardiography, N (%)	52 (3.4)	18 (3.2)	0.854
Bronchoscopy, N (%)	135 (8.7)	40 (7.1)	0.234
Fundoscopy, N (%)	26 (1.7)	12 (2.1)	0.491
Source control			
Source control indicated, N (%)	816 (52.7)	248 (44.0)	< 0.001
Source control attempted, N (%)	795 (94.2)	237 (90.8)	0.056
Source control accomplished, N (%)	680 (83.3)	190 (76.6)	0.017
Other therapeutic measures			
Adequate antibiotic therapy within 24 h, N (%)	825 (53.3)	273 (48.5)	0.051
Corticosteroid therapy, N (%)	389 (25.4)	137 (24.6)	0.715
Renal replacement therapy at onset of BSI, N (%)	288 (18.6)	104 (18.5)	0.945
Extracorporeal membrane oxygenation, N (%)	29 (1.9)	1 (0.2)	0.020
Clinical status and management on day #7			
Renal replacement therapy, N (%)	224 (14.5)	69 (12.3)	0.190
Extracorporeal membrane oxygenation, N (%)	20 (1.3)	0	NA
Glasgow Coma Scale, median (IQR)	13 (9,15)	11 (8,15)	0.005
Mental state, N (%)			
Conscious and normal neurological status	438 (38.0)	133 (33.7)	0.128
Hyporeactive delirium	102 (8.8)	44 (11.1)	0.178
Mixed delirium	45 (3.9)	12 (3.0)	0.434
Hyperreactive delirium	26 (2.3)	8 (2.0)	0.790
Comatose / unconscious, with ongoing sedation	357 (30.9)	123 (31.1)	0.940
Comatose / unconscious, without ongoing sedation	186 (16.1)	75 (19.0)	0.189
Response to treatment			
Resolution, N (%)	331 (28.6)	89 (22.5)	0.019
Improvement, N (%)	547 (47.2)	197 (49.7)	0.380
Clinical failure, N (%)	197 (17.0)	81 (20.5)	0.122
Indeterminate, N (%)	84 (7.2)	29 (7.3)	0.960
Alive on day #7, N (%)	1161 (75.1)	397 (70.5)	0.034
Clinical status on day #28			
Discharged, N (%)	312 (20.2)	67 (11.9)	< 0.001
All-cause mortality, N (%)	520 (33.6)	281 (49.9)	< 0.001
All-cause mortality (excluding individuals whose death was preceded by a decision to withhold or withdraw life-sustaining treatment)^b^, N (%)	374 (26.8)	200 (41.8)	< 0.001

*BSI* bloodstream infection, *IQR* interquartile range (first and third quartiles)

^a^Calculated using logistic regression models adjusted for center effect through generalized estimation equations

^b^A total of 77 (27%) older adults and 142 (27%) younger patients who died by day 28 were excluded from this analysis

### Outcomes

Older adults had higher all-cause mortality at 28-days (50% vs 34% among non-elderly individuals, respectively, *p* < 0.001), and lower discharge rate at 28-days (12% vs 20%, *p* < 0.001). Following exclusion of those whose death was preceded by a decision to avoid life-sustaining treatment (77 (27%) older adults and 142 (27%) younger patients who died by day 28), all-cause mortality remained significantly higher among older adults (42% vs 27%, *p* < 0.001).

### Sensitivity analyses

Following the exclusion of 278 (13.2%) of the cohort who were admitted due to COVID-19, differences in BMI, diabetes mellitus and presentation with non-sedative coma became insignificant. Older adults appeared to have higher proportions of peptic ulcer disease (6% vs 3%, *p* = 0.019) and respiratory source for BSI (29% vs 23%, *p* = 0.014) (Supplementary Table S1).

While response to therapy at 7 days remained slightly better among younger individuals, the all-cause mortality rate at 7 days did not differ between the groups. Yet, age group-related differences in the all-cause mortality rates at 28 days remained unchanged (Supplementary Table S2).

A total of 1183/2111 (56%) patients in 142 (65%) ICUs were from countries with an HDI greater than the median of the cohort. By restricting the analysis to include only these individuals, age group-related disparities in a few baseline characteristics were identified. Moreover, this analysis revealed that among older adults hospitalized in the ICU with BSI, the proportion of admissions due to COVID-19 was lower (7% vs 12%, *p* = 0.025). They also had higher proportion of urinary source for their BSI (10% vs 6%, *p* = 0.028) (Supplementary Table S4). Nonetheless, all-cause mortality at 28 days and source control rates remained unchanged. Yet, all-cause mortality rates at 7 days and discharge rates at 28 days become statistically insignificant. However, as seen in the entire cohort, among older adults, source control less often indicated (49% vs 56%, *p* = 0.023) and, when attempted, was less frequently effective (78% vs 86%, *p* = 0.038) (Supplementary Table S5).

Analysis according to baseline physical status (any functional limitation vs none), regardless of age, revealed that individuals with functional limitation were more likely to undergo CT scan (43% vs 37%, *p* = 0.003). Moreover, among individuals with any limitation, source control was less often indicated (48% vs 54%, *p* = 0.011), pursued (92% vs 95%, *p* = 0.011) or successfully accomplished (79% vs 85%, *p* = 0.013). Individuals with physical limitation were more likely to receive corticosteroid therapy (28% vs 21%, *p* = 0.001) and renal replacement therapy (21% vs 16%, *p* = 0.006) for sepsis and were less likely to undergo ECMO (1% vs 3%, *p* = 0.002). Among older adults, no differences in management were observed between independent individuals and those with functional limitation, except for a higher proportion of corticosteroid therapy among non-independent patients (27% vs 18%, *p* = 0.021) (Supplementary Table S6).

## Discussion

We provided a comprehensive global picture on differences between critically ill individuals ≥ 75 years with younger ones with HA-BSI. We found higher 28-day mortality and lower discharge rate among older adults. Multiple studies reported worse outcomes in older patients admitted to ICU and an increased long-term mortality along with accelerated functional decline [8]. Management was overall similar in both groups with several differences. Older adults were less likely to receive adequate antibiotic therapy within 24 h, though this finding did not reach statistical significance. Importantly, source control was less often indicated among elderly, likely due to lower rates of catheter-related and intra-abdominal source. It also tended to be less attempted and less frequently effective. Delayed drainage and increased age were independent factors for mortality among patients with obstructive pyelonephritis in a previous study [9]. However, source control type and timing also depend on the patient’s conditions, including frailty, nutritional, cognitive, and immunological status. These likely contribute to slower control of sepsis in older patients [10].

In line with current literature, older adults more often had a respiratory or urinary source of HA-BSI. Their higher rates of an unknown source could be explained by difficulties in diagnosing the cause of sepsis among elderly [11]. At HA-BSI, older patients had a higher proportion of coma unrelated to sedative agents, and lower heart rate, blood pressure and temperature. This confirms the teaching that older patients with sepsis often present with altered mental status and apyrexia. They are also more frequently treated with antiarrhythmic and antihypertensive drugs [11]. Considering that CRP monitoring is useful also in the older population [12], it is not surprising that markers of inflammation (leukocytes, CRP, and procalcitonin) at presentation were similar between the groups, and likewise the index of severity (i.e., SOFA score).

The proportion of individuals who died after a decision to forgo life-sustaining therapies was similar in both age groups and paralleled to a previous report [13]. This finding likely reflects background comorbidities of the younger age group. Limitations of these data are lack of information regarding end-of-life decisions and management in different centers.

Our study is somewhat limited by the absence of thorough assessment of frailty using a validated measure, such as the clinical frailty scale [14]. A distinction between frail and non-frail older adults could have refined the role of age and its associations with clinical outcomes following HA-BSI.

Additionally, there is no well-established definition for older adults. We classified our cohort using an age cut-off of 75 years, assuming it carries the best discernment ability. This was also supported by previous literature [2]. However, a different age cut-off would have probably yielded somewhat different results. Additionally, the cohort population was heterogeneous, allowing only descriptive analysis.

In summary, older adults with BSI hospitalized in the ICU had higher comorbidity score, and lower functional capacity compared to younger patients; source of infection differed, and presentation was more atypical in older adults. Management differed mainly in lower rates of source control accomplishment among older adults, and prognosis was poorer. Considering the high mortality from HA-BSI, and the fact that most of them were acquired in the ICU, it is imperative to implement infection prevention measures aiming at diminishing the incidence of HA-BSIs among older adults.

## Supplementary Information

Below is the link to the electronic supplementary material.

Supplementary file 1 (DOCX 102 KB)

Supplementary file 2 (DOCX 23 KB)

## Data Availability

The datasets used and analyzed during the current study are available from the corresponding author on reasonable request.
