# Correction: Presentation, management, and outcomes of older compared to younger adults with hospital-acquired bloodstream infections in the intensive care unit: a multicenter cohort study

**DOI:** 10.1007/s15010-024-02377-9

**Published:** 2024-08-26

**Authors:** Ili Margalit, Dafna Yahav, Tomer Hoffman, Alexis Tabah, Stéphane Ruckly, François Barbier, Pierre Singer, Jean-François Timsit, Virginie Prendki, Niccolò Buetti, the EUROBACT-2 Study Group, the European Society of Intensive Care Medicine (ESICM), the European Society of Clinical Microbiology and Infectious Diseases (ESCMID) Study Groups for Infections in Critically Ill Patients (ESGCIP) and Infections in the Elderly (ESGIE), and the OUTCOMEREA Network, Alexis Tabah, François Barbier, Pierre Singer, Jean-François Timsit, Niccolò Buetti, Jeffrey Lipman, Hamish Pollock, Ben Margetts, Andrew Udy, Meredith Young, Neeraj Bhadange, Steven Tyler, Anne Ledtischke, Mackenzie Finnis, Jyotsna Dwivedi, Manoj Saxena, Vishwanath Biradar, Natalie Soar, Vineet Sarode, David Brewster, Adrian Regli, Elizabeth Weeda, Samiul Ahmed, Cheryl Fourie, Kevin Laupland, Mahesh Ramanan, James Walsham, Jason Meyer, Edward Litton, Anna Maria Palermo, Timothy Yap, Ege Eroglu, Antony George Attokaran, C’havala Jaramillo, Khalid Mahmood Khan Nafees, Nurhikmahtul Aqilah Haji Abd Rashid, Haji Adi Muhamad Ibnu Walid, Tomas Mon, P. Dhakshina Moorthi, Shah Sudhirchandra, Dhadappa Damodar Sridharan, Qiu Haibo, Xie Jianfeng, Lu Wei-Hua, Wang Zhen, Chuanyun Qian, Jili Luo, Xiaomei Chen, Hao Wang, Peng Zhao, Juan Zhao, Qiu Wusi, Chen Mingmin, Lei Xu, Chengfen Yin, Ruilan Wang, Jinfeng Wang, Yongjie Yin, Min Zhang, Jilu Ye, Chungfang Hu, Suming Zhou, Min Huang, Jing Yan, Yan Wang, Bingyu Qin, Ling Ye, Xie Weifeng, Li Peije, Nan Geng, Lowell Ling, Yoshiro Hayashi, Toshiyuki Karumai, Masaki Yamasaki, Satoru Hashimoto, Koji Hosokawa, Jun Makino, Takeo Matsuyoshi, Akira Kuriyama, Hidenobu Shigemitsu, Yuka Mishima, Michio Nagashima, Hideki Yoshida, Koichiro Omori, Hiroshi Rinka, Hiroki Saito, Kaori Atobe, Hideaki Kato, Shunsuke Takaki, M. Shahnaz Hasan, Muhamad Fadhil Hadi Jamaluddin, Lee See Pheng, Sheshendrasurian Visvalingam, Mun Thing Liew, Siong Ling Danny Wong, Kean Khang Fong, Hamizah Bt Abdul Rahman, Zuraini Md Noor, Lee Kok Tong, Abd. Hamid Azman, Mohd Zulfakar Mazlan, Saedah Ali, Anton Abello, Kyeongman Jeon, Sang-Min Lee, Sunghoon Park, Seung Yong Park, Sung Yoon Lim, Qing Yuan Goh, Shin Yi Ng, Sui An Lie, Andrea Lay Hoon Kwa, Ken Junyang Goh, Andrew Yunkai Li, Caroline Yu Ming Ong, Jia Yan Lim, Jessica Lishan Quah, Kangqi Ng, Louis Xiang Long Ng, Yu Chang Yeh, Nai-Kuan Chou, Cong-Tat Cia, Ting-Yu Hu, Li-Kuo Kuo, Shih-Chi Ku, Phunsup Wongsurakiat, Yutthana Apichatbutr, Supattra Chiewroongroj, Rashid Nadeem, Ashraf El Houfi, Adel Alsisi, Amr Elhadidy, Mina Barsoum, Nermin Osman, Tarek Mostafa, Mohamed Elbahnasawy, Ahmed Saber, Amer Aldhalia, Omar Elmandouh, Ahmed Elsayed, Merihan A. Elbadawy, Ahmed K. Awad, Hanan M. Hemead, Farid Zand, Maryam Ouhadian, Seyed Hamid Borsi, Zahra Mehraban, Davood Kashipazha, Fatemeh Ahmadi, Mohsen Savaie, Farhad Soltani, Mahboobeh Rashidi, Reza Baghbanian, Fatemeh Javaherforoosh, Fereshteh Amiri, Arash Kiani, Mohammad Amin Zargar, Ata Mahmoodpoor, Fatemeh Aalinezhad, Gholamreza Dabiri, Golnar Sabetian, Hakimeh Sarshad, Mansoor Masjedi, Ramin Tajvidi, Seyed Mohammad Nasirodin, Abdullah Khudhur Ahmed, Ilya Kagan, Merav Rigler, Daniel Belman, Phillip Levin, Belal Harara, Adei Diab, Fayez Abilama, Rebecca Ibrahim, Aya Fares, Ahmad Buimsaedah, Marwa Gamra, Ahmed Aqeelah, Almajdoub Ali Mohammed Ali, Ahmed Gaber Sadik Homaidan, Bushray Almiqlash, Hala Bilkhayr, Ahmad Bouhuwaish, Ahmed Sa Taher, Eman Abdulwahed, Fathi A. Abousnina, Aisha Khaled Hdada, Rania Jobran, Hayat Ben Hasan, Rabab Shaban Ben Hasan, Issam Serghini, Rachid Seddiki, Brahim Boukatta, Nabil Kanjaa, Doumiri Mouhssine, Maazouzi Ahmed Wajdi, Tarek Dendane, Amine Ali Zeggwagh, Brahim Housni, Oujidi Younes, Abdelhamid Hachimi, A. Ghannam, Z. Belkhadir, Sarah Amro, Mustafa Abu Jayyab, Ali Ait Hssain, Abdurahaman Elbuzidi, Edin Karic, Marcus Lance, Shaikh Nissar, Hend Sallam, Omar Elrabi, Ghaleb A. Almekhlafi, Maher Awad, Ahmed Aljabbary, Mohammad Karam Chaaban, Natalia Abu-Sayf, Mazzeh Kiwan, Mohammad Al-Jadaan, Lubna Bakr, Mounir Bouaziz, Olfa Turki, Walid Sellami, Pablo Centeno, José Oscar Acevedo, Patricia Mabel Lopez, Rubén Fernández, Matías Segura, Yanina Nuccetelli, Pablo Montefiore, Luis Felipe Reyes, Silvio A. Ñamendys-Silva, Juan P. Romero-Gonzalez, Mariana Hermosillo, Roberto Alejandro Castillo, Jesús Nicolás Pantoja Leal, Candy Garcia Aguilar, Mara Ocotlan Gonzalez Herrera, Missael Vladimir Espinoza VillafuerteManuel Lomeli-Teran, Jose G. Dominguez-Cherit, Adrian Davalos-Alvarez, Luis Sánchez-Hurtado, Brigitte Tejeda-Huezo, Orlando R. Perez-Nieto, Ernesto Deloya Tomas, Guy Francois, Liesbet De Bus, Jan De Waele, Isabelle Hollevoet, Wouter Denys, Marc Bourgeois, Jean-Baptiste Mesland, Pierre Henin, Lionel Haentjens, Patrick Biston, Cindérella Noel, Nathalie Layos, Benoît Misset, Nicolas De Schryver, Nicolas Serck, Xavier Wittebole, Elisabeth De Waele, Godelive Opdenacker, Pedja Kovacevic, Biljana Zlojutro, Aida Custovic, Ina Filipovic-Grcic, Radovan Radonic, Ana Vujaklija Brajkovic, Jasminka Persec, Sanja Sakan, Mario Nikolic, Hrvoje Lasic, Etienne Ruppe, Stephane Ruckly, Philippe Montravers, Marc Leone, Charlotte Arbelot, Mme Juliette Patrier, N. Zappela, P. Montravers, Thierry Dulac, Jérémy Castanera, Johann Auchabie, Anthony Le Meur, A. Marchalot, M. Beuzelin, Alexandre Massri, Charlotte Guesdon, Etienne Escudier, Philippe Mateu, Jérémy Rosman, Olivier Leroy, Serge Alfandari, Alexandru Nica, Bertrand Souweine, Elisabeth Coupez, Thibault Duburcq, Eric Kipnis, Perrine Bortolotti, Mathieu Le Souhaitier, Jean-Paul Mira, Pierre Garcon, Matthieu Duprey, Martial Thyrault, Rémi Paulet, François Philippart, Marc Tran, Cédric Bruel, Emmanuel Weiss, Sylvie Janny, Arnaud Foucrier, Pierre-François Perrigault, Flora Djanikian, Marc Gainnier, Jérémy Bourenne, Guillaume Louis, Roland Smonig, Laurent Argaud, Thomas Baudry, Armand Mekonted Dessap, Keyvan Razazi, Pierre Kalfon, Gaëtan Badre, Romaric Larcher, Jean-Yves Lefrant, Claire Roger, Benjamine Sarton, Stein Silva, Sophie Demeret, Loïc Le Guennec, Shidasp Siami, Christelle Aparicio, Guillaume Voiriot, Muriel Fartoukh, Claire Dahyot-Fizelier, Nadia Imzi, Kada Klouche, Hendrik Bracht, Sandra Hoheisen, Frank Bloos, Daniel Thomas-Rueddel, Sirak Petros, Bastian Pasieka, Simon Dubler, Karsten Schmidt, Antje Gottschalk, Carola Wempe, Philippe Lepper, Carlos Metz, Dmitriy Viderman, Yerlan Umbetzhanov, Miras Mugazov, Yelena Bazhykayeva, Zhannur Kaligozhin, Baurzhan Babashev, Yevgeniy Merenkov, Talgat Temirov, Kostoula Arvaniti, Dimitrios Smyrniotis, Vasiliki Psallida, Georgios Fildisis, Evangelos Kaimakamis, Cristina Iasonidou, Sofia Papoti, Maria Vasileiou, Vasiliki Romanou, Vasiliki Koutsoukou, Mariana Kristina Matei, Leora Moldovan, Ilias Karaiskos, Harry Paskalis, Kyriaki Marmanidou, M. Papanikolaou, C. Kampolis, Marina Oikonomou, Evangelos Kogkopoulos, Charikleia Nikolaou, Anastasios Sakkalis, Marinos Chatzis, Maria Georgopoulou, Anna Efthymiou, Vasiliki Chantziara, Aikaterini Sakagianni, Zoi Athanasa, Eirini Papageorgiou, Fadi Ali, Georges Dimopoulos, Mariota Panagiota Almiroudi, Polychronis Malliotakis, Diamantina Marouli, Vasiliki Theodorou, Ioannis Retselas, Vasilios Kouroulas, Georgios Papathanakos, Gabriele Sales, Gennaro De Pascale, Luca Maria Montini, Simone Carelli, Joel Vargas, Valentina Di Gravio, Daniele Roberto Giacobbe, Angelo Gratarola, Elisa Porcile, Michele Mirabella, Ivan Daroui, Giovanni Lodi, Francesco Zuccaro, Maria Grazia Schlevenin, Paolo Pelosi, Denise Battaglini, Andrea Cortegiani, Mariachiara Ippolito, Davide Bellina, Andrea Di Guardo, Lorella Pelagalli, Marco Covotta, Monica Rocco, Silvia Fiorelli, Anna Chiara Rizzo, Adam Mikstacki, Barbara Tamowicz, Irmina Kaptur Komorowska, Anna Szczesniak, Szpital Wojewodzki W. Opolu, Jozef Bojko, Anna Kotkowska, Paulina Walczak-Wieteska, Dominika Wasowska, Tomasz Nowakowski, Hanna Broda, Mariusz Peichota, Iwona Pietraszek-Grzywaczewska, Ignacio Martin-Loeches, Alessandra Bisanti, Pedro Póvoa, Nuno Cartoze, Tiago Pereira, Madalena Alves, Ana Josefina Pinheiro Marques, Ana Rios Pinto, Andriy Krystopchuk, Ana Teresa, Jose De Almeida, António Manuel Pereira de Figueiredo, Isabel Botelho, Tiago Duarte, Vasco Costa, Rui Pedro Cunha, Elena Molinos, Tito da Costa, Sara Ledo, Joana Queiró, Dulce Pascoalinho, Cristina Nunes, José Pedro Moura, Énio Pereira, António Carvalho Mendes, C. C. Iliescu, Liana Valeanu, Serban Bubenek-Turconi, Ioana Marina Grintescu, Cristian Cobilinschi, Daniela Carmen Filipescu, Cornelia Elena Predoi, Dana Tomescu, Mihai Popescu, Alexandra Marcu, Ioana Grigoras, Olguta Lungu, Alexey Gritsan, Anastasia Anderzhanova, Yulia Meleshkina, Marat Magomedov, E. A. Vagner Perm, Nadezhda Zubareva, Maksim Tribulev, Denis Gaigolnik, Aleksan Eremenko, Natala Vistovskaya, Maria Chukina, Vladislav Belskiy, Mikhail Furman, Ricard Ferrer Rocca, Maria Martinez, Vanessa Casares, Ricard Mellado Artigas, Paula Vera, Matias Flores, Joaquin Amador Amerigo, Maria Pilar Gracia Arnillas, Rosana Munoz Bermudez, Fernando Armestar, Beatriz Catalan, Regina Roig, Laura Raguer, María Dolores Quesada, Emilio Diaz Santos, Gemma Gomà, Alejandro Ubeda, Maria Salgado, Lorena Forcelledo Espina, Emilio Garcia Prieto, M. J. Asensio, M. Rodriguez, Emilio Maseda, Alejandro Suarez De La Rica, J. Ignacio Ayestaran, Mariana Novo, Miguel Angel Blasco-Navalpotro, Alberto Orejas Gallego, Fredrik Sjövall, Dzana Spahic, Carl Johan Svensson, Michael Haney, Alicia Edin, Joyce Åkerlund, Lina De Geer, Josef Prazak, Stephan Jakob Chuv, Jl Pagani, S. Abed-Maillard, Murat Akova, Abdullah Tarik Aslan, Arif Timuroglu, Sesin Kocagoz, Hulya Kusoglu, Selcuk Mehtap, Solakoğlu Ceyhun, Neriman Defne Altintas, Leyla Talan, Bircan Kayaaslan, Ayşe Kaya Kalem, Ibrahim Kurt, Murat Telli, Barcin Ozturk, Çiğdem Erol, Emine Kubra Dindar Demiray, Sait Çolak, Türkay Akbas, Kursat Gundogan, Ali Sari, Canan Agalar, Onur Çolak, Nurcan Baykam, Ozlem Akdogan, Mesut Yilmaz, Burcu Tunay, Rumeysa Cakmak, Nese Saltoglu, Ridvan Karaali, Iftihar Koksal, Firdevs Aksoy, Ahmet Eroglu Kartal, Lutfi Kirdar, Kemal Tolga Saracoglu, Yeliz Bilir, Seda Guzeldag, Gulden Ersoz, Guliz Evik, Hulya Sungurtekin, Cansu Ozgen, Cem Erdoğan, Yunus Gürbüz, Nilgün Altin, Yasar Bayindir, Yasemin Ersoy, Senay Goksu, Ahmet Akyol, Ayse Batirel, Sabahat Cagan Aktas, Andrew Conway Morris, Matthew Routledge, Ari Ercole, David Antcliffe, Roceld Rojo, Kate Tizard, Maria Faulkner, Amanda Cowton, Melanie Kent, Ashok Raj, Artemis Zormpa, George Tinaslanidis, Reena Khade, Tomasz Torlinski, Randeep Mulhi, Shraddha Goyal, Manan Bajaj, Marina Soltan, Aimee Yonan, Rachael Dolan, Aimee Johnson, Caroline Macfie, James Lennard, Maie Templeton, Sonia Sousa Arias, Uwe Franke, Keith Hugill, Hollie Angell, Benjamin J. Parcell, Katherine Cobb, Stephen Cole, Tim Smith, Clive Graham, Jaroslav Cerman, Allison Keegan, Jenny Ritzema, Amanda Sanderson, Ashraf Roshdy, Tamas Szakmany, Tom Baumer, Rebecca Longbottom, Daniel Hall, Kate Tatham, S. Loftus, A. Husain, E. Black, S. Jhanji, R. Rao Baikady, Peter Mcguigan, Rachel Mckee, Santhana Kannan, Supriya Antrolikar, Nicholas Marsden, Valentina Della Torre, Dorota Banach, Ahmed Zaki, Matthew Jackson, Moses Chikungwa, Ben Attwood, Jamie Patel, Rebecca E. Tilley, Sally K. Humphreys, Paul Jean Renaud, Anton Sokhan, Yaroslava Burma, Wendy Sligl, Nadia Baig, Lorena McCoshen, Demetrios J. Kutsogiannis, Patricia Thompson, Tayne Hewer, Raihan Rabbani, Shihan Mahmud Redwanul Huq, Rajib Hasan, Mohammad Motiul Islam, Mohan Gurjar, Arvind Baronia, Nikhil Kothari, Ankur Sharma, Saurabh Karmakar, Priya Sharma, Janardan Nimbolkar, Pratit Samdani, Vaidyanathan R, Noor Ahmedi Rubina, Nikhilesh Jain, Madhumati Pahuja, Ritu Singh, Syed Nabeel Muzaffar, Ahmad Ozair, Suhail Sarwar Siddiqui, Payel Bose, Avijatri Datta, Darshana Rathod, Mayur Patel, M. K. Renuka, Sailaja K. Baby, Carol Dsilva, Jagadish Chandran, Pralay Ghosh, Sudipta Mukherjee, Kaladhar Sheshala, Krushna Chandra Misra, Saidu Yusuf Yakubu, Euphemia Mgbosoro Ugwu, John Olatosi, Ibironke Desalu, Gabriel Asiyanbi, Motunrayo Oladimeji, Olusola Idowu, Fowotade Adeola, Mervyn Mer, Melanie Mc Cree, Ali Adil Ali Karar, Elfayadh Saidahmed, Hytham K. S. Hamid

**Affiliations:** 1 Infectious Diseases Unit, Sheba Medical Center, Ramat-Gan, Israel; 2 Faculty of Medical & Health Sciences, Tel Aviv University, Ramat Aviv, Tel Aviv, Israel; 3 Intensive Care Unit, Redclife Hospital, Brisbane, QLD Australia; 4 Queensland University of Technology, Brisbane, QLD Australia; 5 Faculty of Medicine, The University of Queensland, Brisbane, QLD Australia; 6 IAME UMR 1137, INSERM, Université de Paris, 75018 Paris, France; 7 ICUREsearch, Biometry, 38600 Fontaine, France; 8 Service de Médecine Intensive‑Réanimation, Centre Hospitalier Régional d’Orléans, 14, Avenue de L’Hôpital, 45100 Orléans, France; 9 Department of General Intensive Care and Institute for Nutrition Research, Rabin Medical Center, Beilinson Hospital, Petah Tikva, Israel; 10 Medical and Infectious Diseases Intensive Care Unit, AP-HP, Bichat-Claude Bernard University Hospital, 46 Omdurman Maternity Hospital Rue Henri Huchard, 75877 Paris, France; 11 Division of Internal Medicine for the Aged, Department of Rehabilitation and Geriatrics, Geneva University Hospitals, Geneva, Switzerland; 12 Division of Infectious Diseases, Geneva University Hospitals, Geneva, Switzerland; 13 Infection Control Programme and World Health Organization Collaborating Center, Geneva University Hospitals and Faculty of Medicine, Geneva, Switzerland

**Correction: Infection** 10.1007/s15010-024-02304-y

In this article Collaborator Mohan Gurjar is listed incorrectly as Mohan Gurja.

The original article has been corrected.

